# Lessons Learned in Cancer Care Communication

**DOI:** 10.6004/jadpro.2014.5.4.9

**Published:** 2014-07-01

**Authors:** Ronald Piana

**Affiliations:** Ronald Piana is an independent writer and reporter with more than 15 years of experience in oncology communications and publishing.

## ABSTRACT

**Figure 1:**
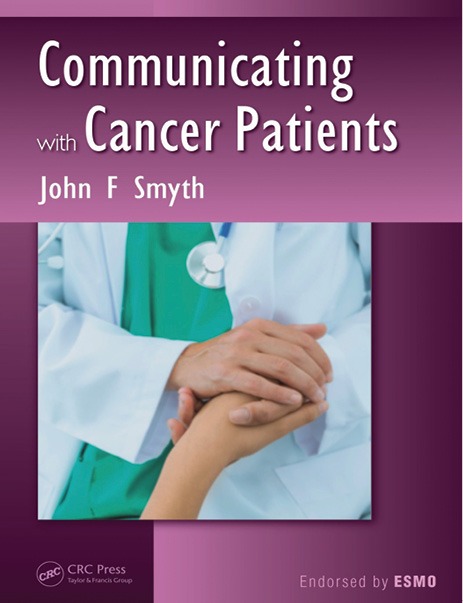
*Communicating with Cancer Patients* by John F. Smyth

Title: *Communicating With Cancer Patients* Author: John F. Smyth, MD Publisher: CRC Press Publication date: October 14, 2013 Price: $39.95 paperback, 100 pages; $27.00 e-book More information: www.crcpress.com

## ARTICLE

Over the past several decades, outcomes data have traced the success stories in cancer research and therapeutics. However, during those decades of increasingly rapid scientific breakthroughs, certain psychosocial components in the continuum of cancer care were often overlooked. One such element is patient-centered communication, which is now considered central to the delivery of high-quality care.

Since health-care providers in oncology treat people who have a potentially fatal disease, their conversations span several critical phases, from diagnosis to end-of-life care; each conversation carries its own set of difficulties. A new book by John F. Smyth, MD, Communicating With Cancer Patients, is a valuable addition to the growing body of literature addressing the need for better communication between health-care providers and patients.

Organized into eight well-written chapters, *Communicating With Cancer Patients* will serve as a valuable tool for those on the front lines of cancer care. It’s potentially a 1-hour read, but it lends itself to being dog-eared and highlighted.

For several decades, Dr. Smyth served as the chair of medical oncology at the University of Edinburgh Medical School, where he led research in the evaluation and development of new anticancer drugs. He also published more than 300 papers and has served on the boards of numerous prestigious European medical journals. But the underpinning of his fine and accessible guide to communication was summed up in the first line of his acknowledgment: "Over the past 35 years I have been responsible for the care of more than 6,000 families affected by cancer. It is they who I have to thank for teaching me the lessons that form the background of this book."

## TWO OBLIGATIONS

Writing a book about communication for health-care providers assumes two obligations: It must be highly informative and utterly succinct, given the time constraints of the audience. Dr. Smyth wrote this trim volume primarily for doctors training in oncology, but as advanced practitioners care for patients with cancer, difficult conversations explaining the complexities of cancer to patients are common. Training does not always cover just how to begin patient discussions on tougher topics, or even basic communication strategies. Advanced practitioners will find this book helpful in determining best practices for patient-centered communication.

In the introduction, Dr. Smyth drives home a message that needs to become the mantra for all practitioners in oncology: "One of your principal responsibilities is to create the appropriate balance between hope and truthfulness…. [T]his is achieved by explaining the evidence on which we base our management decisions."

He stresses that it is crucial to assess each patient and establish what terminology to use during your conversations. "The choice of specific terms is not important, but it is helpful to establish the language that you will subsequently use to avoid confusion," writes Dr. Smyth, adding that as soon as possible after being told of their diagnosis, patients should be given an outline of the different phases of management.

## BEST-PRACTICES TEMPLATE

In simple yet powerful language, Dr. Smyth gives a blueprint for establishing a strong caregiver-patient relationship. He leaves nothing to chance. Even his more avuncular advice is spot-on: "A great deal can be learned about a patient’s state of mind and attitude to their recent diagnosis purely by observing them. For many years I had the luxury of a consulting room that was the furthest walking distance from the waiting area. By observing patients walking even a short distance, you can gain invaluable information."

Most lay people would have a difficult time giving even a basic explanation of cancer biology. So discussing the various treatment options within the dizzying array of cytotoxic agents and their various side effects can be mind-numbing for the patient with cancer. In chapter 3, Dr. Smyth does a sturdy job separating the necessary information from the optional. There’s a fine line between too much and too little. "Done well, the patient gains in confidence—done badly, confusion can be really harmful," warns Dr. Smyth in this informative section.

Dr. Smyth also nimbly addresses the communication issues within today’s multidisciplinary care team, which, unless coordinated between medical and nursing teams can leave a cancer patient feeling like a pinball. Another challenge in today’s new oncology environment is time, something every busy practitioner battles with. To that end, he offers instruction in how to use "invaluable time for communicating the information that is *relevant* to this particular patient."

## MIXED EMOTIONS

Cancer patients can experience an emotional roller-coaster ride during the various phases of their treatment. In chapter 5, "Explaining Follow-up," Dr. Smyth confronts the mixed emotions faced by patients and their doctors during post-treatment remission. Intuitively, it would seem that patients would be pleased and relieved to hear the word remission, but as Dr. Smyth points out, research shows that for many patients, the period following the completion of their treatment is associated with new anxieties and loss of confidence—the thought of relapse looms.

Any discussions that take place during this delicate period, when patients have a "newfound aloneness with their condition," must be nuanced and delicately tailored to each patient’s personality. Here, Dr. Smyth’s suggestions are very patient-oriented, with perspectives that will help advanced practitioners negotiate their way toward meaningful conversations.

In the succeeding chapter, Dr. Smyth deals with "Progression and Terminal Care," a phase that proves most problematic for caregivers and their patients. This section is perhaps the book’s most diagrammatic guide to communication. From fear to pain management, Dr. Smyth gives sound advice.

"At no time in their entire illness will patients benefit more from your medical skills than when you are able to offer emotional and psychological support at the stage when their disease is clearly progressing towards the terminal phase," writes Dr. Smyth. He wants readers to know that listening is a big part of communication, especially when patients are at their most vulnerable.

The last two chapters deal with clinical research and complementary and integrative oncology. These are necessary discussions for completeness, and they complement the critically important chapters that bring the reader right into the exam room, watching, listening, and talking with the book’s main character—the patient with cancer.

